# Indatraline: Synthesis and Effect on the Motor Activity of Wistar Rats

**DOI:** 10.3390/molecules16119421

**Published:** 2011-11-10

**Authors:** Márcia Kameyama, Fernanda A. Siqueira, Miriam Garcia-Mijares, Luiz F. Silva, Maria T. A. Silva

**Affiliations:** 1 Department of Experimental Psychology, Institute of Psychology, University of São Paulo, USP, Av. Prof. Mello de Moraes, 1721, Cidade Universitária, São Paulo SP CEP 05508-030, Brazil; Email: marcia_kameyama@yahoo.com.br (M.K.); mgarciam@usp.br (M.G.-M.); 2 Departamento de Química Fundamental, Instituto de Química, Universidade de São Paulo, CP 26077, São Paulo-SP, 05513-970, Brazil; Email: nandasiq2003@yahoo.com.br (F.A.S.)

**Keywords:** indatraline, cocaine abuse, ring contraction, motor activity, Wistar rats

## Abstract

A new approach for the synthesis of indatraline was developed using as the key step an iodine(III)-mediated ring contraction of a 1,2-dihydronaphthalene derivative. Behavioral tests were conducted to evaluate the effect of indatraline and of its precursor indanamide on the motor activity of Wistar rats. Specific indexes for ambulation, raising and stereotypy were computed one, two and three hours after i.p. drug administration. Indatraline effects on motor activity lasted for at least three hours. On the other hand, no significant differences in motor activity were observed using indanamide. The results suggest that indatraline has a long lasting effect on motor activity and add evidence in favor of the potential use of that compound as a substitute in cocaine addiction.

## 1. Introduction

**Scheme 1:**
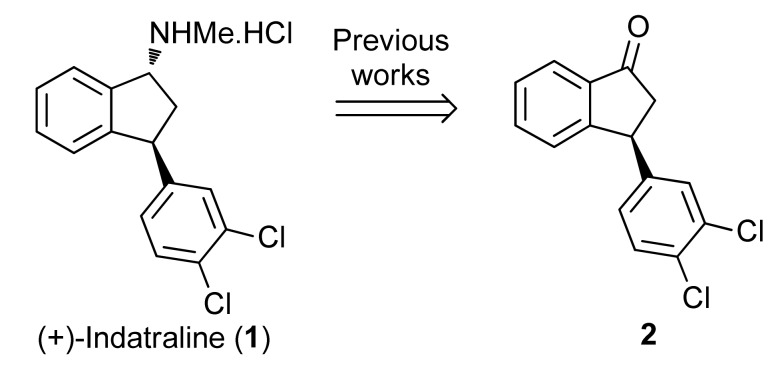
Previous approaches toward indatraline.

One pharmacological approach to cocaine abuse treatment relies on the development and use of compounds that target the dopamine (DA) transporter. Indatraline (**1**, Scheme 1) is a non-selective monoamine reuptake inhibitor [1], acting on the DA transporter with effects similar to those of cocaine, but with slower onset and longer duration [2]. As such, it is a potential candidate for treatment of cocaine abuse [3]. Although there is no evidence in favor of clinical use of indatraline in cocaine addiction [4], a cocaine-like chemical profile has prompted systematic animal studies investigating its potential as a substitute agonist medication. The concept of substitute agonist therapy, successfully used in nicotine and heroin addictions, implies that the substitute compound will show some cocaine-like characteristics. Most important among these would be reinforcing properties and psychomotor stimulant effects.

Some studies suggest that indatraline may have mild reinforcing properties. In fact, it has been shown that the discriminative effects of indatraline are qualitatively similar to those of cocaine in self-administration procedures in monkeys [5] and in rats [6]. Thus, an adequate indatraline dose could prove to be a complete substitute for cocaine. Although producing undesirable side-effects, indatraline doses produced a sustained decrease in cocaine self-administration during several days of treatment [5]. In rats, however, indatraline failed to show consistently this effect, but produced modest effects on reinstatement of extinguished cocaine response [7]. State discrimination studies have shown that rats [8] and monkeys [5] were able to discriminate indatraline from vehicle effects after being trained on a cocaine *versus* vehicle discrimination. Tirelli and Witkin [9] focused stereotypical gnawing behavior, demonstrating that indatraline is more potent than cocaine in inducing this behavior in mice.

Since the ideal substitute for cocaine should achieve a subtle balance between induced cocaine-like effects and reduced drug use, it seems that most results so far reported do not fully satisfy this condition. One important gap regards motor activity. Even though this variable is considered a good predictor of reinforcing activity [10,11] and cocaine is a classic psychomotor stimulant, there are no specific studies of indatraline’s effect on motor activity.

Regarding chemical syntheses, previous studies to obtain the *trans*-3-aryl-1-amino-indane ring system of indatraline and analogues relied on the preparation of a 1-indanone, which was transformed into the target 3-phenyl-1-indanamine through classic reactions [12,13,14,15,16,17,18] One asymmetric synthesis of the more active (+)-indatraline [12] was reported from **2**, using this approach (Scheme 1) [19].

**Figure 1:**
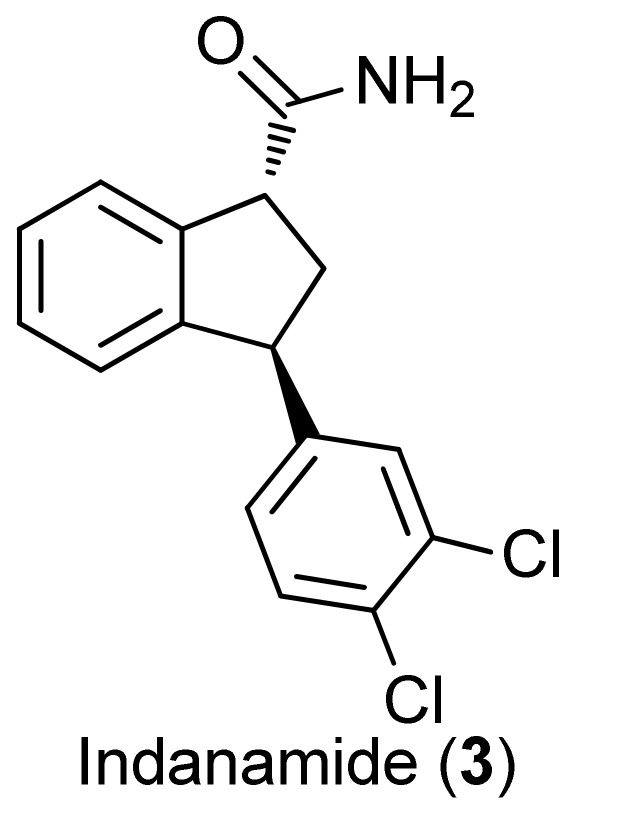
Structure of indanamide (**3**).

Considering the above scenario, we started a research program aiming at: (i) a new and efficient route to prepare indatraline in sufficient amounts for biological evaluation, *i.e.*, nearly one gram [20]. In this novel approach hypervalent iodine reagents [21,22,23,24,25,26] are used in the key transformations; (ii) the general and specific motor activity of both (±)-indatraline and its structural analogue indanamide (**3**) (Figure 1) were also evaluated.

## 2. Results and Discussion

### 2.1. Diastereoselective Synthesis of Indatraline

**Scheme 2:**
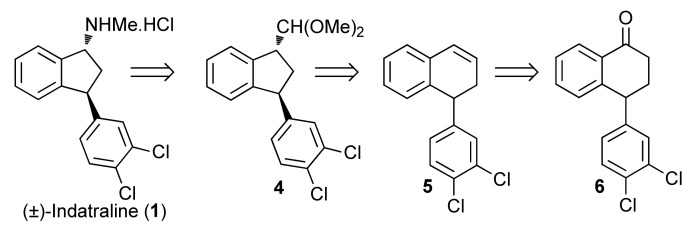
Retrosynthesis for (±)-indatraline (**1**) using a ring contraction approach.

We envisioned that (±)-indatraline (**1**) could be efficiently obtained from the *trans*-1,3-disubstituted indane **4**, using a Hofmann rearrangement promoted by I(III) and functional group transformations. The indane **4** would be formed through an I(III)-mediated ring contraction of the 1,2-dihydronaphthalene **5**, which would be prepared by classical reactions from the known tetralone **6** (Scheme 2). The ketone **6** is produced in an optically pure form on industrial scale, because it constitutes the starting material of the anti-depressive (+)-sertraline, one of the top selling drugs [27]. Thus, the adaptation of our approach to the synthesis of (+)-indatraline would be possible.

**Scheme 3:**
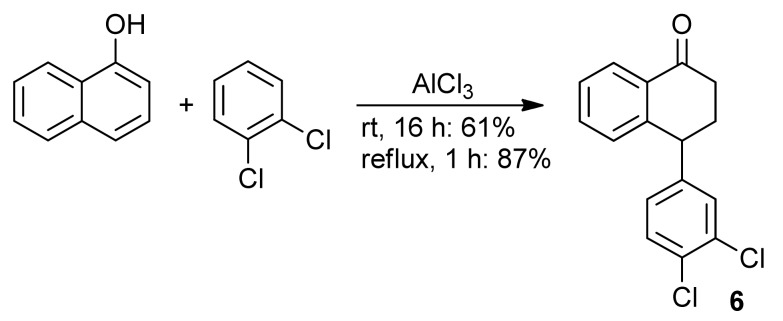
Preparation of **6** from α-naphtol.

**Scheme 4:**
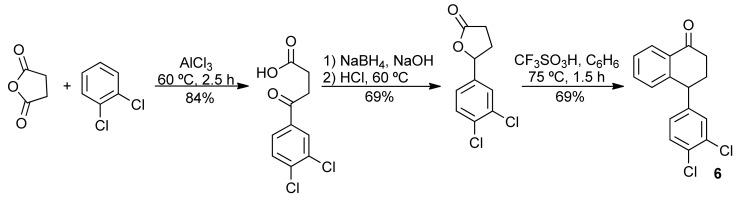
Preparation of **6** from succinic anhydride.

**Scheme 5:**
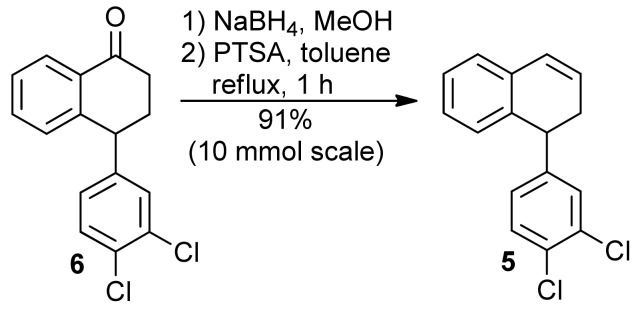
Synthesis of 1,2-dihydronaphthalene **5**.

The tetralone **6** was obtained from α-naphthol and 1,2-dichlorobenzene [28] (Scheme 3) and from 1,2-dichlorobenzene and succinic anhydride in three steps (Scheme 4) [29]. Next, the tetralone **6** was reduced with NaBH_4_, giving the corresponding 1-tetralol. The dehydration of this alcohol was best performed with PTSA, which furnished the desired 1,2-dihydronaphthalene **5**, in 91% yield (Scheme 5). When the dehydration was performed with H_3_PO_4_, the 1,2-dihydronaphthalene was isolated in good yield only on a small scale. Using more than 1 mmol of starting material **6**, the formation of a complex mixture was observed [30].

The oxidation of 1,2-dihydronaphthalene **5** with iodine(III) [20,22,31,32,33,34] or with thallium(III) [30,31,33,35,36,37,38,39,40] in MeOH furnished the indane **4**, the naphthalene **7** and the addition product **8**, depending on the reaction conditions (Table 1). With 0.9 equiv. of HTIB the reaction afforded **4** in only 25% yield. The naphthalene **7** and a mixture of diastereomers **8** were also isolated (entry 1). A higher amount of HTIB was used to make the reaction faster and, consequently, to avoid formation of naphthalene. When the reaction was performed with 3.6 equiv. of HTIB in anhydrous MeOH, the indane **4** was obtained in 62% yield, as a single diastereomer, together with the addition product **8**, in 35% yield (entry 4). With a lower amount of HTIB, the yield of the ring contraction product **4** is smaller (entries 1–3). A similar pattern was also observed in Tl(III) reactions, where an excess of the oxidant increased the yield of the indane [41]. The reaction was performed in MeOH 95% to investigate the importance of the use of anhydrous solvent. Under these conditions, a smaller yield was obtained when compared with the reaction performed in anhydrous MeOH (entries 5 and 6). The ring contraction product was also obtained using thallium(III) trinitrate (TTN). Treatment of the olefin **5** with TTN in MeOH lead to the indane **4** in 80% yield (entry 7). When the solvent was substituted for trimethyl orthoformate (TMOF), **4** was obtained in 88% yield (entry 8). To prepare the required amount of **4** to progress the synthesis, we use the best condition with iodine(III) (entry 4) to avoid the generation of residues of toxic thallium salts.

**Table 1:** Oxidation of 1,2-dihydronaphthalene **5** with HTIB in MeOH. 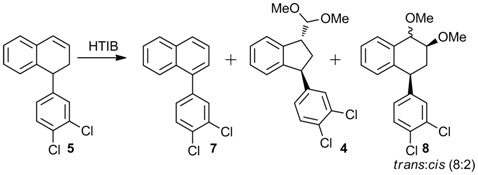

Entry	HTIB	MeOH	Temperature	Time	Products (Yield)
1	0.9 equiv.	anyd	r.t.	24 h	**7** (17%); **4** (25%); **8** (26%).
2	1.4 equiv.	anyd	r.t.	3.5 h	**7** (35%); **8** (39%).
3	2.8 equiv.	anyd	r.t.	1 h	**4** (50%); **8** (39%).
4	3.6 equiv.	anyd	r.t.	15 min	**4** (62%); **8** 35%.
5	1.2 equiv.	95%	r.t.	30 min	**4** (16%); **8** (9%)
6	2.0 equiv.	95%	r.t.	30 min	**4** (26%); **8** (35%)
7	1.1 equiv. TTN	95%	r.t.	2 min	**4** (80%)
8	1.1 equiv. TTN	TMOF	0 °C	5 min	**4** (88%)

**Scheme 6:**
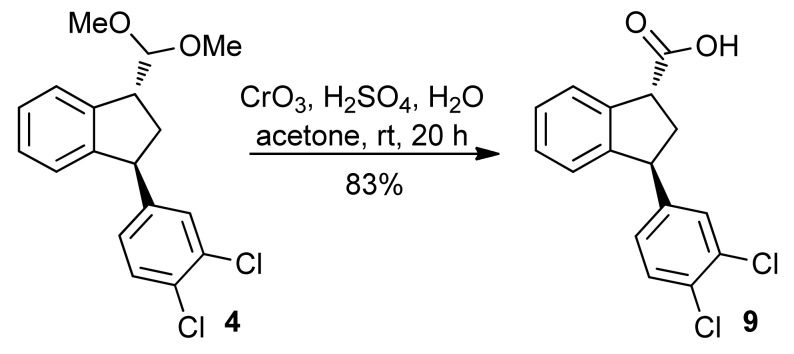
Preparation of carboxylic acid **9**.

The ketal moiety of **4** was oxidized using Jones’ reagent (CrO_3_/H_2_SO_4_ in acetone) giving directly the carboxylic acid **9** in 83% yield (Scheme 6). Presumably, the aldehyde moiety is first unmasked and through its hydrate is oxidized to **9**. X-ray analysis of the crystalline solid **9** clearly shows the *trans* relationship of the substituents in the cyclopentane ring [20].

**Scheme 7:**
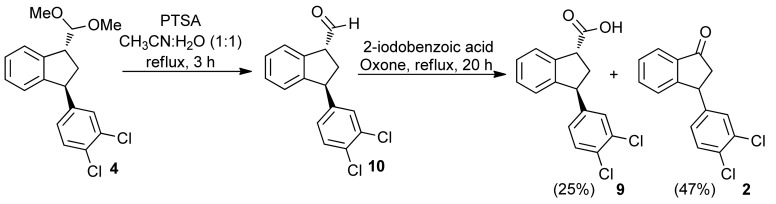
Reaction of **10** with IBX.

The preparation of carboxylic acid **9** was also performed with IBX [42,43] as oxidant to avoid the use of a heavy metal. The ketal **4** was hydrolyzed with catalytic amount of PTSA in CH_3_CN:H_2_O (1:1) furnishing the corresponding aldehyde **10**, which was reacted with IBX without isolation. The IBX was generated *in situ* from 2-iodobenzoic acid and oxone^®^ [44,45,46]. Under this condition, the indane **9** was obtained in only 25% yield, because the major product was the indanone (±)-**2**, which was isolated in 47% yield (Scheme 7).

**Scheme 8:**
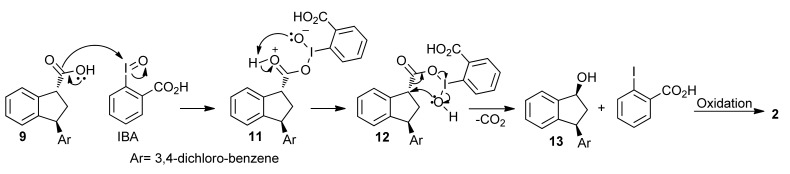
Mechanism for the formation of **2**.

The formation of indanone **2** may occur by the oxidative decarboxylation of **9**, promoted by I(III). The nucleophilic addition of carboxylic acid **9** to IBA would furnish the intermediate **11**. The oxyanion **11** would abstract the hydrogen of the hydroxyl group with consequent stabilization of the positive charge on oxygen. The rearrangement of the intermediate **12** would lead to the indanol **13** which would be oxidized to indanone **2** by IBX (Scheme 8). Alternatively, the formation of ketone 2 could occur by the following sequence of events: (i) α-hydroxylation of aldehyde **10** promoted by an iodine(III) species present in the reaction medium; (ii) hydration of the aldehyde moiety; and (iii) oxidative cleavage of the vicinal diol moiety promoted by IBX [47].

**Scheme 9:**
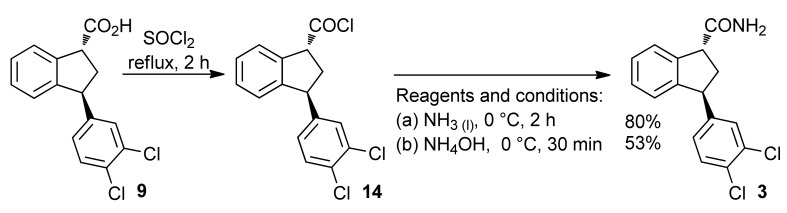
Preparation of amide **3**.

Using classical conditions, the acid **9** was converted into amide **3**. Higher yields were obtained using liquid ammonia than an aqueous solution of NH_4_OH (Scheme 9). X-ray analysis of the crystalline solid **3** shows the *trans*-relationship of the cyclopentane ring [48].

**Scheme 10:**
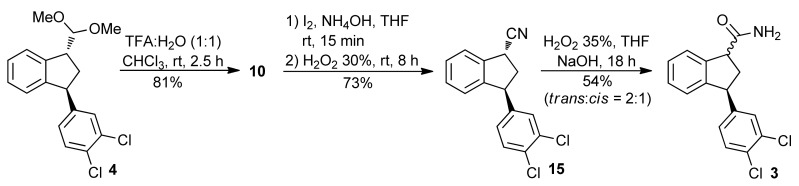
Preparation and hydrolysis of **15**.

We also envisioned the preparation acid giving the aldehyde **10** in 81% yield, after purification. The compound **10** was then of amide **3** by reaction of nitrile **15** with H_2_O_2_ [49], thus avoiding the use of CrO_3_. The ketal **4** was hydrolyzed with trifluoroacetic reacted with iodine and NH_4_OH at room temperature, followed by treatment of a solution of H_2_O_2_, giving the nitrile **15** in 73% yield. This nitrile was converted into amide **3** using H_2_O_2_ under basic conditions. However, the epimerization of the amide **3** was observed, giving the *trans*:*cis* isomers in a ratio of 2:1, respectively (Scheme 10).

**Scheme 11:**
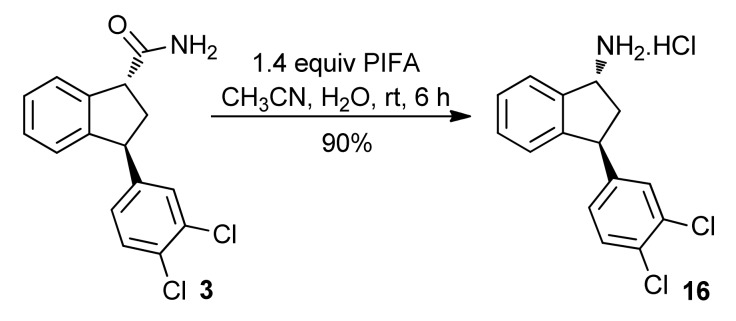
Hofmann rearrangement of amide **3**.

The required Hofmann rearrangement of the amide **3** was performed by the hypervalent iodine reagent PhI(OCOCF_3_)_2_ (PIFA) [50], cleanly gave the primary amine **16**, in the form of the corresponding hydrochloride (Scheme 11). This compound is also crystalline, which allowed the elucidation of the structure by X-ray analysis [20] The amino and the aryl group are in a *trans* relationship in the cyclopentane ring, which shows that the rearrangement occurred with retention of configuration as expected.

**Scheme 12:**
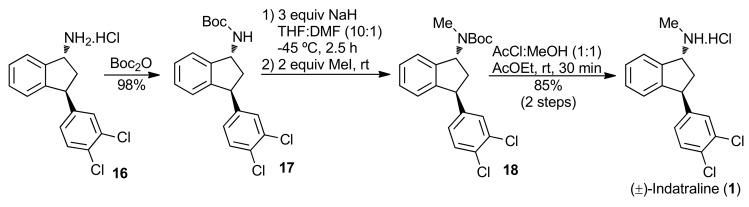
Final steps toward indatraline (**1**).

To conclude the synthesis, the primary amine **16** should be transformed into the corresponding secondary methylamine. After testing several protocols, we found that indatraline could be obtained from **16** in three steps. The first step was the protection of the amine with the Boc group to yield the corresponding carbamate **17** [51], which was alkylated with MeI leading to **18** [52,53]. A careful adjust of the reaction conditions was necessary. The use of 3.0 equiv. of NaH in a mixture of THF-DMF (10:1) at −45 °C was found to be the optimal conditions for the alkylation step. When this condition was not followed, epimerization was observed. After treatment of **18** with HCl generated *in situ * [54] indatraline (**1**) was obtained as a crystalline solid (Scheme 12). Many tests were also performed toward the reduction of **17** using hydride sources (LiAlH_4_ and Red-Al^®^) directly to (±)-**1**. Reductive amination protocols were also tried using **16** as substrate. However, in all these tests only complex mixtures were obtained and/or starting material was recovered.

Using the route described above, we were able to prepare nearly one gram of indatraline (**1**), as well as enough quantities of indanamide (**3**). This material was used in the experiments described in the next section.

### 2.1. Motor Activity of Indatraline (**1**) and Indanamide (**3**)

#### 2.1.1. Experiment 1

**Figure 2:**
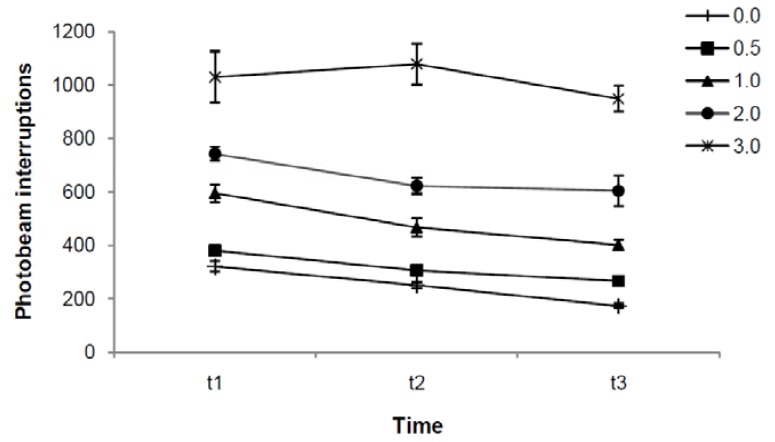
Effect of indatraline on total motor activity. Data represent means (±SE) of photobeam interruptions during 60 (t1), 120 (t2) and 180 (t3) minutes after administration of 0.0, 0.5, 1.0, 2.0 and 3.0 mg/kg. ANOVA indicated that indatraline dose and time were independently related to motor activity measures. Bonferroni post-hoc comparisons revealed time-dependent decreases in motor activity and differences between 2.0 and 3.0 mg/kg when compared to vehicle.

Figure 2 shows total motor activity as a function of time after administration and indatraline dose. Drug dose [F(4,35) = 18.43, p = 0.000] and time [F(2,70) = 16.91; p = 0.000] were independently related to motor activity measures [*i.e*., no interaction between these factors was found: F(8,70) = 1.06, p = 0.403], suggesting that dose-dependent drug effects were stable over time. Bonferroni post-hoc comparisons revealed time-dependent decreases in motor activity (t1 *vs.* t2 p = 0.012; t1 *vs.* t3 p = 0.000 and t2 *vs.* t3 p = 0.017). They also revealed that 2.0 and 3.0 mg/kg (p = 0.003 and p = 0.000, respectively) but not 0.5 and 1.0 mg/kg of indatraline (p = 1.000 and p = 0.238, respectively) enhanced total motor activity when compared to vehicle (0.0 mg/kg).

To better understanding the effects of indatraline on motor activity a MANOVA for drug dose and time effects on three motor activity topographies (ambulation, stereotypy and raising) was performed. MANOVA results indicated interactions between topography and dose [F(8,68) = 7.59, p = 0.000] and between topography and time [F(4,32) = 6.65, p = 0.001], but not between dose and time [F(8,68) = 1.26, p = 0.277] or between these three factors [F(16,98) = 1.23, p = 0.256]. So, topography differences related to time and dose effects of indatraline were found, but dose effects on motor topographies were not dependent on time.

**Figure 3:**
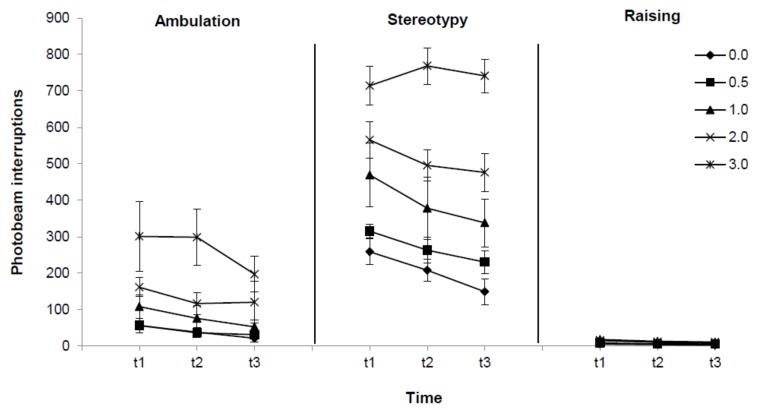
Effect of indatraline (**1**) on ambulation, stereotypy and raising episodes. Data represent means (±SE) of photobeam interruptions during 60 (t1), 120 (t2) and 180 (t3) minutes after drug administration of 0.0, 0.5, 1.0, 2.0 and 3.0 mg/kg) for each activity topography (ambulation, stereotypy and raising episodes). MANOVA showed that drug dose and time effects were dependent on topography. Bonferroni *post-hoc* comparisons indicated that stereotypy was enhanced by 1.0, 2.0 and 3.0 mg/kg of indatraline, ambulation was affected by 3.0 mg/kg and raising was unaffected by the drug. Time-dependent changes on motor activity were observed for ambulation and stereotypy, but not for raising.

As shown in Figure 3, Bonferroni *post-hoc* comparisons for dose and topography suggested that, compared to vehicle, stereotypy behavior was enhanced by administration of 1.0 (p = 0.016), 2.0 (p = 0.000) and 3.0 mg/kg (p = 0.000) indatraline, but not by 0.5 mg/kg (p = 1.000). Conversely, indatraline effect on ambulation was only observed at the highest dose (0.0 *vs.* 3.0 mg/kg p = 0.001) and had no effect at any dose on raising responses (p = 1.000 for all comparisons between each dose and vehicle). Time-dependent effects are also indicated in Figure 3. As seen in that Figure, differences on stereotypy were detected between t1 and t3 (p = 0.000) but not between t1 and t2 (p = 0.075) or between t2 and t3 (p = 0.289). Differences in ambulation were also observed between t1 and t3 (p = 0.005), but not between t1 and t2 (p = 1.000) or between t2 and t3 (p = 1.000). Finally, no time-dependent differences in raising were found (p = 1.000 for all comparisons between times).

#### 2.1.2. Experiment 2

**Figure 4:**
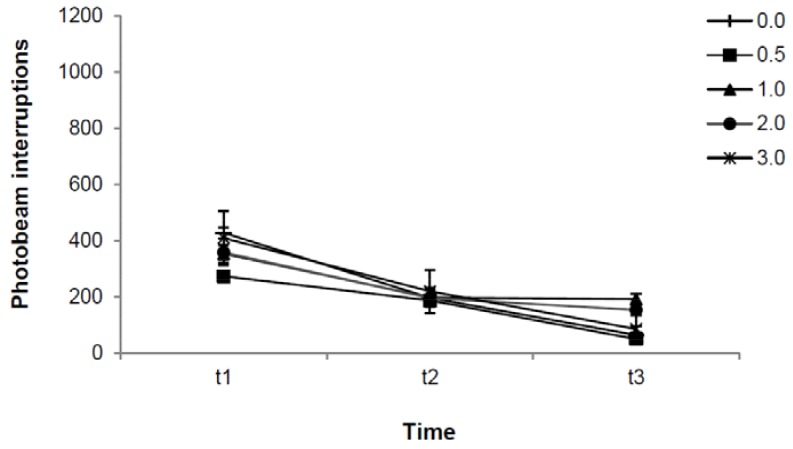
Effect of indanamide (**3**) on total motor activity. Data represent means (±SE) of photobeam interruptions during 60 (t1), 120 (t2) and 180 (t3) minutes after administration of 0.0, 0.5, 1.0, 2.0 and 3.0 mg/kg. ANOVA indicated that time but not indanamide **3** had a significant effect on motor activity. Bonferroni *post-hoc* test revealed time-dependent decreases on motor activity.

Effects of indanamide (**3**) dose and time after administration on total motor activity are shown in Figure 4. Mixed ANOVA results indicated that time [F(2,70) = 58.06, p = 0,000], but not indanamide (**3**) dose [F(4,35) = 2.36; p = 0.072) had a significant effect on motor activity. The analysis suggested non-significant interaction effects between dose and time [F(8,70) = 1.60; p = 0.141]. *Post-hoc* Bonferroni tests for time revealed time-dependent decreases on motor activity (t1 *vs. *t2 p = 0.000; t1 *vs.* t3 p = 0.000 and t2 *vs.* t3 p = 0.001).

**Figure 5:**
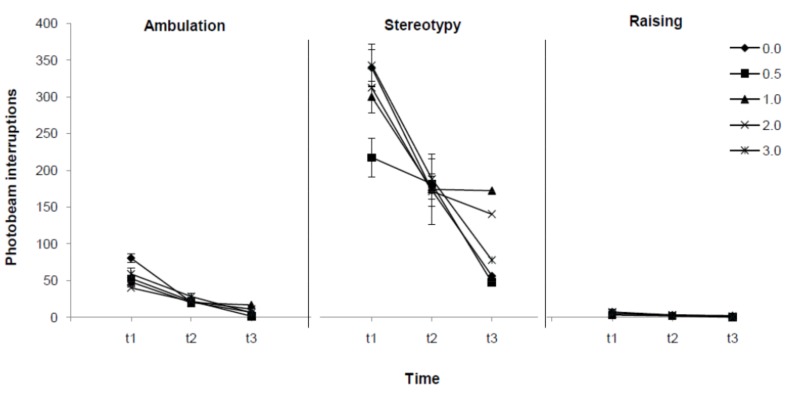
Effect of indanamide (**3**) on ambulation, stereotypy and raising episodes. Data represent means (±SE) of photobeam interruptions during 60 (t1), 120 (t2) and 180 (t3) minutes after administration of 0.0, 0.5, 1.0, 2.0 and 3.0 mg/kg for each activity topography (ambulation, stereotypy and raising episodes). MANOVA showed that time, but not drug effects, was dependent on topography. Bonferroni *post-hoc* test indicated that stereotypy and ambulatory episodes decreased over time.

Data for time and indanamide (**3**) dose effects on the three motor activity topographies (ambulation, stereotypy and raising) are shown in Figure 5. MANOVA results revealed significant interactions between motor topography and time [F(4,32) = 24.81, p = 0.000], but not between motor topography and drug dose [F(8,68) = 1.91, p = 0.072] or between these three factors [F(16,98) = 1.41, p = 0.153]. Bonferroni *post-hoc* test for motor topography and time suggested that stereotypy behavior decreased over time (t1 *vs.* t2 p=0.000; t2 *vs.* t3 = 0.000 and t1 *vs.* t3 p = 0.000). On the other hand, ambulatory activity decreased from t1 to t2 and t3 (p = 0.048 and p = 0.000, correspondingly), but was stable from t2 to t3 (p = 1.000). Raising activity did not change throughout time (p = 1.000 for all comparisons).

#### 2.1.3. Discussion

As a potential candidate for cocaine abuse substitute therapy, the indirect monoamine reuptake inhibitor indatraline (**1**) was tested for its capacity to alter activity of rats. Similarly to cocaine, the DA indirect agonist increased motor activity, specifically the ambulation and stereotypy topographies.

Differently from cocaine, the effect of indatraline on motor activity decreased only slightly (about 8%) three hours after administration. Data obtained by Maisonneuve *et al.* [55] showed that cocaine (20 mg/kg i.p.) enhancing effect on motor activity dropped by almost 60% two hours after administration and had virtually vanished by 3 h. So, it seems that indatraline’s effects on motor activity are quite persistent, lasting perhaps more than those of cocaine. A similar relation was obtained by Rosenzweig-Lipson *et al.* [2] who showed that indatraline’s effects on bar-pressing were more persistent than those produced by cocaine. Indanamide (**3**) was obtained for the first time during our chemical synthesis of indatraline [20]. Thus, no information about the biological properties of compound **1** was available in the literature. Our data show that indanamide (**3**), a precursor of indatraline, was inactive as far as motor activity is concerned.

Motor and rewarding effects of stimulant drugs have been shown to be correlated [56] and are probably mediated by the same dopaminergic pathways [10,11]. Together with data indicating that indatraline produces a mild reinforcing effect in monkeys as evaluated by self-administration [5] and functions as a discriminative stimulus for cocaine-trained animals [5,8] as well as displays a slower onset and longer duration than cocaine and thus may be less likely to be abused [5,57,58] the present results add to the evidence supporting the potential use of indatraline as a substitute in cocaine addiction.

Indatraline potential for treating cocaine dependence should be taken with caution, since although at present there are no human studies on the clinical efficacy of indatraline, its potential for treating cocaine dependence should be further investigated due to the current evidence supporting the clinical use of DA agonists to this end [59,60]. However, animal data such as these presented here are crucial, because assessing the efficacy of pharmacotherapy is a time-consuming task that should only be undertaken in case of promising evidence.

## 3. Experimental

### 3.1. Synthesis of Indatraline (**1**)

#### 3.1.1. General Information

HTIB and PIFA were used as received. Toluene and hexanes were distilled from sodium wire and stored in a bottle also containing sodium wire. MeOH, acetone, CH_3_CN, AcOEt, were distilled from magnesium turnings, KMnO_4_, CaH_2_ and CaH_2_, respectively. DMF was distilled from toluene. These solvents were stored in a bottle containing 4 Å molecular sieves. THF and CH_2_Cl_2_ were freshly distilled from sodium/benzophenone and CaH_2_, respectively. Column chromatography was performed using 200–400 mesh silica gel. TLC analysis were performed using silica gel plates, using phosphomolibidic acid, vaniline or *p*-anisaldehyde solution for visualization. ^1^H- and ^13^C-NMR spectra were recorded on Bruker or Varian spectrometers. IR spectra were measured on a Perkin-Elmer 1750-FT. Gas chromatography analysis were performed in a HP-6890 series II and/or Shimadzu-2010. Melting points are uncorrected. HRMS analysis were performed on a Bruker Daltonics Microtof Electrospray instrument. Experimental procedures and characterization data not mentioned in the article can be found in the preliminary communication [20].

#### 3.1.2. Deprotection of Aldehyde **10** and Its Reaction with 2-Iodobenzoic acid and Oxone

To a stirred solution of ketal **4** (0.0314 g, 0.0931 mmol) in CH_3_CN-H_2_O (1:1, 1.5 mL), were added a few crystals of PTSA. The mixture was refluxed for 3 h. The mixture was cooled to the room temperature and 2-iodobenzoic acid (9.20 mg, 0.0371 mmol) and oxone (0.0744 g, 0.121 mmol) were added. The mixture was refluxed for 20 h. The organic phase was extracted with EtOAc, washed with saturated solution of NaHCO_3_, with H_2_O, with brine, and dried over anhydrous MgSO_4_. The solvent was removed under reduced pressure, giving a yellow oil that was purified by flash chromatography (gradient elution, 30–70% EtOAc in hexanes). Indanone **2** [17,19] (0.0120 g, 0.0433 mmol, 46%) was obtained as a colorless oil and acid **9** [20] (0.0070 mg, 0.0228 mmol, 25%) as a white solid.

#### 3.1.3. 1,3-trans-3-(3,4-Dichlorophenyl)-2,3-di-hydro-1H-indene-1-carbaldehyde (**10**)

To a stirred solution of ketal **4** (0.107 g, 0.317 mmol) in CHCl_3_ (1.0 mL) was added a solution of TFA (50% in H_2_O, 0.5 mL) at 0 °C. The mixture was stirred for 30 min and for 2 h at rt. The reaction was quenched by slow addition of a saturated solution of NaHCO_3_ at 0 °C. The mixture was extracted with CHCl_3_, washed wish saturated solution of NaHCO_3_, with H_2_O, with brine, and dried over anhydrous Na_2_SO_4_. The solvent was removed under reduced pressure, giving a yellow oil that was purified by flash chromatography (20% EtOAc in hexanes). The aldehyde **10** (0.0748 g, 0.257 mmol, 81%) was obtained as a colorless oil. Only NMR analysis was performed and the aldehyde was used in the next step without further analysis to avoid decomposition. ^1^H-NMR (300 MHz, CDCl_3_): δ 2.22 (dt, *J *= 13.5 and 8.4 Hz, 1H), 3.02 (ddd, *J *= 13.5, 8.4 and 3.2 Hz, 1H), 4.08–4.11 (m, 1H), 4.43 (t, *J *= 8.4 Hz, 1H), 6.98–7.01 (m, 2H), 7.24–7.25 (m, 1H), 7.27–7.30 (m, 1H), 7.31–7.34 (m, 1H), 7.38 (d, *J *= 8.1 Hz, 1H), 7.41–7.44 (m, 1H), 9.72 (d, *J *= 2.1 Hz, 1H). ^13^C-NMR (75 MHz, CDCl_3_): δ 36.0, 49.5, 57.0, 125.2, 125.7, 127.4, 127.8, 128.7, 129.9, 130.6, 130.7, 132.7, 138.4, 145.0, 146.2, 199.3.

#### 3.1.4. 1,3-trans-3-(3,4-Dichlorophenyl)-2,3-dihydro-1H-indene-1-carbonitrile (**15**)

To a stirred solution of aldehyde **10** (0.0459 g, 0.158 mmol) and I_2_ (0.0440 g, 0.173 mmol) in THF (0.2 mL), was added an aqueous solution of NH_3_ (28%, 1.0 mL) at rt. After 15 min the color of the reaction mixture changed from black to pale yellow. A solution of H_2_O_2_ (30%, 0.15 mL) was added. The mixture was stirred for 8 hours and extracted with EtOAc. The organic phase was washed with H_2_O, with brine, and dried over anhydrous MgSO_4_. The solvent was removed under reduced pressure and the crude product was purified by flash chromatography (10% EtOAc in hexanes), giving the nitrile **15** (0.0336 g, 0.116 mmol, 73%) as an orange oil. IR (film): 2238, 2940, 3027, 3070 cm^−1^. ^1^H-NMR (300 MHz, CDCl_3_): δ 2.43 (ddd, *J* = 13.5, 8.4 and 7.1 Hz, 1H), 2.90 (ddd, *J* = 13.2, 8.1 and 5.1 Hz, 1H), 4.26 (dd, *J* = 8.4 and 5.1 Hz, 1H), 4.58 (t, *J* = 7.2 Hz, 1H), 6.94 (dd, *J* = 2.1 Hz, 1H), 7.03–7.07 (m, 1H), 7.18 (d, *J* = 2.1 Hz, 1H), 7.30–7.36 (m, 3H), 7.49–7.52 (m, 1H). ^13^C-NMR (75 MHz, CDCl_3_): δ 33.4, 40.7, 48.9, 120.4, 124.8, 125.6, 127.2, 128.4, 129.4, 129.7, 130.7, 131.2, 132.9, 137.6, 143.3, 144.5. LRMS *m/z*: (%): 288 (M^+•^, 23), 262 (10), 141 (100). HRMS *m/z* C_16_H_11_Cl_2_N (M + Na)^+^ calcd. 310.0161, found 310.0161.

### 3.2. Materials and Methods

#### 3.2.1. Animals

The work described in this article was carried out in accordance with EC Directive 2010/63/EU for animal experiments. Male naïve Wistar rats (N = 80) weighing 350–400 g (Instituto Butantan, São Paulo, Brazil) were housed individually in transparent polyethylene homecages (25 × 40 × 20 cm). The lights in the colony room were on from 7:00 a.m. to 7:00 p.m. During the experiment food and water were freely available.

#### 3.2.2. Drugs

Indatraline (**1**) was dissolved in 0.9% NaCl and indanamide (**3**) in dimethyl sulfoxide 50%. All drugs or vehicle were injected i.p. in a volume of 1.0 mL/kg.

#### 3.2.3. Apparatus

Activity tests were conducted in two identical Plexiglas activity chamber (432 × 432 × 305 mm) manufactured by Med Associates (ENV-515, St. Albans, VT, USA). An array of three 16 × 16 × 16 photodetectors, spaced 25 mm apart, was used to detect motor activity.

#### 3.2.4. Procedures

Two experiments were performed. In each experiment forty rats were randomly assigned to five groups (n = 8). On test day animals from each group were injected with either vehicle (0.0 mg/kg) or one of four doses (0.5, 1.0, 2.0 and 3.0 mg/kg) of indatraline (**1**) (Experiment 1) or indanamide (**3**) (Experiment 2), as described in Table 2. On the test day, rats were taken from the colony room and transported to the experimental room, where they remained undisturbed for a 10-min acclimatization period to the room. Next, each animal received a vehicle or drug injection (see Table 2) and was immediately placed in the activity chamber for three hours. Each animal was tested once.

**Table 2:** Treatments for each group in Experiment 1 and Experiment 2.

Experiment	Drug	Group/Dose
1	Indatraline	mg/kg (8)
		0.5 mg/kg (8)
		1.0 mg/kg (8)
		2.0 mg/kg (8)
		3.0 mg/kg (8)
2	Indanamide	0.0 mg/kg (8)
		0.5 mg/kg (8)
		1.0 mg/kg (8)
		2.0 mg/kg (8)
		3.0 mg/kg (8)

#### 3.2.5. Statistical Analysis

Activity measures were obtained using Med-Associates Activity Monitor (ver. 4.31) software configured with resolution (rate of acquisition of the data from the test chambers) set to 50 ms. Three activity topographies were analyzed: stereotypy, ambulation and raising. Stereotypy episodes were defined as movements made within an area defined by four beams (“stereotypy area”). When the subject stayed within this area for 500 ms beam breaks were counted as stereotypy episodes, but when the animal left the stereotypy area during this period, beam breaks were counted as ambulation or raising episodes. Ambulation and raising episodes were counted when at least three horizontal (ambulation) or vertical (raising) beams were interrupted. Total motor activity was obtained by adding stereotypy, raising and ambulation episodes. Measures taken during the first hour (t1), second hour (t2) and third hour (t3) were analyzed by STATISTICA 8.0 (Statsoft Inc., Tulsa, OK, USA) software. Total motor activity was examined by mixed ANOVA using drug dose (0.0, 0.5, 1.0, 2.0 and 3.0 mg/kg) as between-subjects factor and time (t1, t2, t3) as within factor. Results from motor activity topographies (ambulation, stereotypy and raising) were evaluated by mixed MANOVA, in which drug dose was used as between-subjects factor and time (t1, t2, t3) as within factor. *Post-hoc* tests were performed using Bonferroni’s method.

## 4. Conclusions

In conclusion, (±)-indatraline (**1**) was synthesized in nine steps from the readily available tetralone **6**, in 29% overall yield. This new approach features two diastereoselective rearrangements promoted by iodine(III), exemplifying the importance of hypervalent iodine in the synthesis of biologically active compounds. Finally, the described route may be adapted to the asymmetric version starting from (+)-tetralone **6**. Indatraline (**1**), a non-selective monoamine reuptake inhibitor, significantly increased motor activity in a long-lasting manner. No major differences in motor activity were observed for indanamide (**3**), an indatraline precursor. It is suggested that, due to its behavioral profile, indatraline may have a potential use as a substitute in cocaine addiction. Further research should target, for example, the effect of indatraline on cocaine withdrawal or drug-seeking models.
